# Clinically significant association between the maximum standardized uptake value on ^18^F-FDG PET and expression of phosphorylated Akt and S6 kinase for prediction of the biological characteristics of renal cell cancer

**DOI:** 10.1186/s12885-015-1097-0

**Published:** 2015-03-10

**Authors:** Tomoya Mizuno, Takao Kamai, Hideyuki Abe, Setsu Sakamoto, Kazuhiro Kitajima, Daisaku Nishihara, Hideo Yuki, Tsunehito Kambara, Hironori Betsunoh, Masahiro Yashi, Yoshitatsu Fukabori, Yasushi Kaji, Ken-Ichiro Yoshida

**Affiliations:** 1 Department of Urology, Dokkyo Medical University, 880 Kitakobayashi Mibu, Tochigi, 321-0293 Japan; 2 PET Center, Dokkyo Medical University Hospital, Mibu, Tochigi, Japan; 3 Department of Radiology, Dokkyo Medical University, Mibu, Tochigi, Japan

**Keywords:** Renal cell carcinoma (RCC), Positron emission tomography (PET), [^18^F]fluorodeoxyglucose (^18^F-FDG), Maximum standardized uptake value (SUVmax), Akt, S6 ribosomal protein

## Abstract

**Background:**

The relationship between the clinicopathological features and molecular changes associated with standardized uptake value (SUV) determined by Positron emission tomography (PET) with [18F] fluorodeoxyglucose (18F-FDG PET) in human renal cell carcinoma (RCC) has not been elucidated. On the other hand, overactivation of the phosphatidylinositol 3’kinase (PI3K), serine/threonine kinase Akt, and mammalian target of rapamycin (mTOR) pathway has been detected in a variety of human cancers, including RCC. So far, little is known about the relationship between the SUV and these proteins in human RCC. Thus, it is important to study the relevance of SUV with clinicopathological features in human RCCs from a molecular point of view.

**Methods:**

Seventy-seven consecutive patients with RCC who underwent nephrectomy and pretreatment determination of the maximum SUV (SUVmax) by 18F-FDG PET were analyzed. We investigated the relationship between the SUVmax, phosphorylated-Akt (Ser-473) (pAkt(Ser-473)), phosphorylated-Akt (Thr-308) (pAkt(Thr-308), and phosphorylated-S6 ribosomal protein (Ser-235/236) (pS6) protein levels in the primary tumor and various clinicopathological features.

**Results:**

The average SUVmax of the primary tumor was 6.9 (1.5 to 40.3). A higher SUVmax was correlated with higher expression of pAkt(Ser-473), pAkt (Thr-308), and pS6 protein in the primary tumor. A higher SUVmax and increased expression of pAkt (Ser-473), pAkt (Thr-308), and pS6 of the primary tumor was associated with less tumor differentiation, a higher pT stage, regional lymph node involvement, microscopic vascular invasion, and distant metastasis, as well as with early relapse following radical nephrectomy in patients who had localized or locally advanced RCC without distant metastasis (cTanyNanyM0) and with shorter overall survival in all patients.

**Conclusions:**

A higher SUVmax on 18F-FDG PET is associated with elevated tumor levels of pAkt and pS6 protein and with aggressive behavior and metastatic potential of RCC, as well as with early relapse following radical nephrectomy and shorter overall survival. These findings suggest that SUVmax may be useful for predicting the biological characteristics of RCC.

## Background

Renal cell carcinoma (RCC) is the most common solid cancer of the kidney and accounts for about 3% of adult malignancies. RCC is one of the top 10 causes of cancer deaths in industrialized countries and its incidence has consistently increased over the past few decades [1]. Localized RCC is usually treated surgically, but almost 30% of patients with limited disease at the time of resection develop metastasis within 3 years [2,3]. Clear cell RCC is a type of renal cancer that is extremely vascular with a high metastatic potential, and a high percentage of patients with clear cell RCC already have metastases at diagnosis or else relapse after nephrectomy [2,3]. Metastasis involves the spread of tumor cells from the primary lesion to a distant site [4], and it is the major cause of cancer death. RCC patients with distant metastasis have a poor prognosis and their 5-year survival rate is less than 10% [2,3].

Increased glucose uptake is a key alteration associated with the high glycolytic rate of cancer cells (the Warburg effect) [5], and reprogramming of energy metabolism can now be viewed as one of the “hallmarks of cancer” [6]. Oxidative phosphorylation is impaired in RCC, resulting in a metabolic shift to aerobic glycolysis [7]. The phosphatidylinositol 3‘kinase (PI3K), serine/threonine kinase Akt, and mammalian target of rapamycin (mTOR) pathway is abnormally active in a number of human cancers, including RCC [8], and interruption of this pathway by targeted therapy has antiproliferative, pro-lethal, antiangiogenic, and pro-apoptotic effects, and are more effective against RCC than previous cytokine therapy or chemotherapy [9].

Positron emission tomography (PET) can be performed using [^18^F]fluorodeoxy-glucose (^18^F-FDG PET) to assess fundamental alterations of cellular glucose metabolism [10]. The maximum standard glucose uptake value (SUVmax) is the most common semiquantitative parameter determined by ^18^F-FDG PET and a decrease of SUVmax is associated with the response to numerous anticancer therapies [11]. ^18^F-FDG PET has been suggested as a noninvasive pharmacodynamic marker for the response to novel targeted anticancer agents in patients with advanced RCC [12-14]. It has been shown that inhibition of Akt disrupts transcription of glucose transporter protein-1 (GLUT1) and its translocation to the plasma membrane to promote glucose utilization independent of an effect on cell proliferation [15]. Thus, it has been proposed that ^18^F-FDG PET could be used as a pharmacodynamic biomarker for assessing the efficacy of inhibition of the PI3K/Akt/mTOR pathway by targeted therapy [10]. However, little is known about the relationship between the clinicopathological features and molecular changes associated with SUVmax in human RCCs. To our knowledge, this is the first reported study in which we examine the relationship between SUVmax and phosphorylated-Akt (Ser-473) (pAkt (Ser-473), phosphorylated-Akt (Thr-308) (pAkt (Thr-308), and phosphorylated-S6 ribosomal protein (Ser-235/236) (pS6) expression levels in human RCC. Our findings might lead to new insight on the value of SUVmax as a tumor biomarker in RCC from a molecular point of view.

## Methods

### Patients and samples

We studied 77 consecutive Japanese patients (48 men and 29 women) aged from 39 to 83 years (mean age: 63.1 years) in whom RCC was diagnosed from 2010 to 2013. All patients underwent ^18^F-FDG PET/computed tomography (CT) for preoperative staging prior to radical nephrectomy. The postoperative follow-up period ranged from 3 to 45 months (median: 19 months). Surgery was performed before patients received any other therapy. The patient profile and tumor characteristics are summarized in Table 1. The pretreatment SUVmax on ^18^F-FDG PET was defined as baseline SUVmax. In every patient, three tumor tissue specimens and various non-neoplastic kidney tissues were harvested and stored at −80°C as soon as possible after nephrectomy, as described previously [16,17]. The non-neoplastic kidney tissues were apparently free of tumor cells and were obtained from as far distant a part of the resected kidney as possible. If the tumor was located centrally, non-neoplastic tissues were harvested from the upper or lower pole, while non-neoplastic tissues were harvested from the opposite pole if the tumor was located in the upper or lower pole. The tumor grade and clinical stage were determined according to the Fuhrman system and TNM classification, respectively [18,19]. Histopathological examination of the resected kidneys was performed independently by two pathologists. If abnormalities of the putatively normal tissue samples were detected, the patient was excluded from the study. In the present study, however, in all 77 patients, non-neoplastic tissues resected were confirmed to be non-cancerous tissues by microscopic examination. This study was conducted in accordance with the Helsinki Declaration and was approved by the ethical review board of Dokkyo Medical University Hospital. Each patient signed a consent form that was approved by our institutional Committee on Human Rights in Research.

**Table 1:** **Data collection of patient and tumor characteristics**

Patient		
	No. of patients	77
	Age (yrs)	63.1 (39–83)
	Sex (male/female)	48/29
	follow-up times (months)	19 (3–43)
**Tumor**		
	Histology (clear cell/cell/sarcomatoid differentiation)	57/11/9
	Histological Fuhrman grading (G1/G2/G3/G4)	9/32/29/7
	pT stage (T1/T2/T3/T4)	24/19/32/2
	pN stage (T0/N1/N2)	62/12/3
	Microscopic vascular invasion (v0/v1)	28/49
	Metastasis (M0/M1)	52/25
**Surgery**	Radical nephrectomy	77
	with cavotomy with thrombectomy for > T3b tumor	(9)
	with RPLND* for > N1 tumor	(15)

RPLND*: retroperitoneal lymph node dissection.

Postoperative adjuvant therapy with interferon (IFN)-alpha (3, 5, or 6 million units of natural human IFN-alpha two or three times weekly), sorafenib (400 to 800 mg/day), or sunitinib (25 to 50 mg/day for 4 weeks, followed by two weeks off therapy) was usually provided if patients had extra-renal involvement (metastatic disease) and was continued until progression occurred. The doses of these agents were decreased if the patient developed grade 3/4 toxicity.

### Performance and evaluation of ^18^F-FDG PET /CT

Whole-body imaging using a combined ^18^F-FDG PET/CT scanner (Biograph, Sensation 16, Siemens Systems) and evaluation of the images were performed according to our previously reported methods [20,21]. Whole-body CT covered a region ranging from the meatus of the ear to the mid-thigh. The 64-detector-row helical CT scanner had a gantry rotation speed of 0.5 sec, a table speed of 24 mm per rotation, 120 kVp, 40 mA, and a slice thickness of 2.5 mm. The PET imager of the combined system had an axial view of 16.2 cm (per bed position) with an interslice interval of 3.75 mm and FWHM of 5 mm in one bed position. Imaging from the meatus of the ear to the mid-thigh was achieved with six to seven bed positions. The transaxial field of view and pixel size of the PET images reconstructed for fusion was 58.5 cm and 4.57 mm, respectively, with a 128 × 128 matrix. To avoid artifacts caused by the urinary tract, patients were asked to drink 1000 ml of water at 1–2 h prior to image acquisition and to empty the bladder just before the start of imaging. Bladder catheterization was not done and intravenous or oral contrast medium was not used. After fasting for at least 4 h, patients received an intravenous injection of ^18^F-FDG (4.0 MBq/kg). Blood glucose was checked in all patients before performing ^18^F-FDG PET, and no patient had a blood glucose level >160 mg/dl. About 50 min later, CT scanning was conducted and whole-body emission PET scanning was performed with acquisition for 3 min per bed position using the three-dimensional acquisition mode. Attenuation-corrected PET images were reconstructed with an ordered-subset expectation maximization iterative reconstruction algorithm that employed eight subsets and three iterations. Images obtained by ^18^F-FDG PET or CT and fused ^18^F-FDG PET/CT images were generated for review on a workstation (AZE Virtual Place Version 3.0035).

^18^F-FDG PET images and CT scans were interpreted by two experienced radiologists and decisions were made by consensus. ^18^F-FDG PET data were used to reconstruct coronal, axial, and sagittal images as is typically done for clinical assessment. When focal ^18^F-FDG PET uptake with a higher intensity than that of the surrounding tissues was seen at a site unrelated to physiologic/nonpathologic processes, metastatic RCC was suspected. Metastasis was diagnosed when abnormal focal ^18^F-FDG PET uptake on PET images corresponded to an abnormal mass on CT scans. The mean activity of the region of interest (ROI) was calculated (MBq/g)/(injected dose (MBq)/body weight (g)) where MBq is mega-Becquerel and g is grams. The SUV was determined according to the standard formula, with activity in the volume of interest (VOI) being calculated as Bq/ml divided by the injected dose in Bq/kg. In each patient, the average SUV was calculated from all images obtained about 1 h after tracer injection. SUVmax was defined as the maximum activity within the VOI. To obtain the highest SUVmax in patients with multiple metastatic tumors, the values of each lesion detected on CT scans were compared.

### Western blotting

We performed Western blotting of samples from all resected primary tumors. Tumor samples and normal control samples were carefully dissected to remove stromal tissue. In order to allow for inter-individual variation in the expression of pAkt (Ser-473), pAkt (Thr-308), and pS6, tumor tissue samples and the corresponding non-neoplastic samples obtained from the same patient were compared as described previously [16,22]. In brief, 10 μg of cytosolic protein was separated by SDS-PAGE (4-12% gel), and was transferred to a polyvinylidene difluoride membrane (iBlot Gel Transfer Stacks PVDF, Mini; Life Technologies, Carlsbad, CA). After the membrane was blocked, the bound proteins were probed with the following primary antibodies: a rabbit anti-human antibody targeting pAkt (Ser-473) (Cell Signaling Technology, Inc; PhosphoPlus Akt (Ser-473) Antibody Kit; # 9270, Danvers, MA), a rabbit anti-human antibody for pAkt (Thr-308) (Cell Signaling Technology, Inc; Phospho-Akt (Thr308) Antibody Kit; # 2965, Danvers, MA), a rabbit anti-human antibody targeting pS6 (2 F9, Cell Signaling Technology, Inc; # 4856), and an antibody for beta-actin (Millipore; # 1501R Bedford, MA). Hela cells were used as the positive control [16,22]. The membranes were washed and incubated with horseradish peroxidase-conjugated secondary antibodies. After protein bands were visualized by chemiluminescence, each membrane was scanned for densitometry with a PDI imaging scanner (Agfa Japan, Tokyo) and the data were analyzed with NIH Image software (ImageJ for Mac OS, version 1.47). Expression of pAkt (Ser-473), pAkt (Thr-308), and pS6 was calculated relative to that of beta-actin in the tumor tissue specimens and corresponding non-neoplastic specimens. For quantification of protein levels, the relative amount of pAkt (Ser-473), pAkt (Thr-308), and pS6 in tumor tissue specimens was expressed as a ratio of the optical density for the tumor specimen to that for the corresponding non-neoplastic specimen (set at 1.0) by densitometric analysis, as described previously [16,17]. The mean values for tumor and non-neoplastic tissues were calculated from three experiments [16,17].

### Immunohistochemistry

To confirm the data obtained by Western blotting, immunohistochemistry was performed with the same antibodies utilized for Western blotting on representative tumors from the 5 patients, as described previously [23].

### Statistical analysis

Western blotting data were analyzed by the Mann–Whitney *U* test for comparisons between two groups (TNM stage, microscopic vascular invasion, serum CRP, and systemic treatment effect), while the Kruskal-Wallis test was used to compare data on histological grade among three groups [16,17]. Spearman’s rank correlation coefficient analysis was performed to determine the relations between SUVmax and the expression of pAkt (Ser-473), pAkt (Thr-308), or pS6, as well as Karnofsky performance status and tumor size [16,24]. The Kaplan-Meier method was used to estimate survival and differences were assessed by the log-rank test [16,17]. The impact on survival of SUVmax, pAkt (Ser-473), pAkt (Thr-308), and pS6 expression, tumor grade, pT stage, regional lymph node involvement, microscopic vascular invasion, and distant metastasis was assessed by Cox proportional hazards analysis using univariate and multivariate models. In all analyses, a *P* value of less than 0.05 was considered significant. Data were analyzed with commercially available software.

## Results

### SUVmax and expression of pAkt and pS6 in the primary tumor

**Figure 1:**
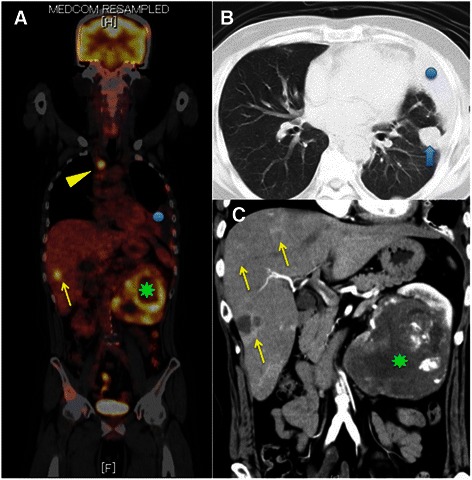
**PET/CT scan.** A case (50 y.o, male) with pT3aN0M1 (HEP, PUL, OSS) with clear cell carcinoma with sarcomatoid differentiation (Fuhrman grade 4). **A**: PET scan shows accumulation of FDG in left renal tumor (green asteroid, SUVmax: 14.5), liver (yellow arrows, SUVmax: 7.1), lung (not shown, SUVmax: 3.7), and Th3 vertebra (yellow arrowhead, SUVmax: 8.8). In this case, the highest SUVmax of the metastatic tumors (m-SUVmax) is 8.8. Blue circle shows left pleural effusion. **B**: Chest CT shows a tumor in left lung (blue arrow) and pleural effusion (blue circle). **C**: Enhanced abdominal CT shows large left renal tumors (green asteroid) and multiple liver tumors (yellow arrows).

All patients had lesions showing increased uptake on FDG-PET/CT in the primary tumor at diagnosis (mean ± S.D of SUVmax = 6.97 ± 5.96, range: 1.5 – 40.3, Figure 1).

**Figure 2:**
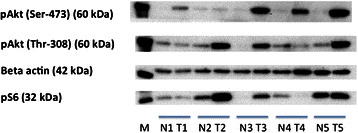
**Expression of phosphorylated Akt (Ser-473) (60 kDa), phosphorylated Akt (Thr-308) (60 kDa), phosphorylated S6 ribosomal protein (Ser-235/236) (32 kDa) and beta actin (42 kDa) proteins in the primary tumor tissues using Western blotting.** M; marker. N; non-tumor tissue. T; primary tumor tissue. Each number corresponds to a case number.

**Figure 3:**
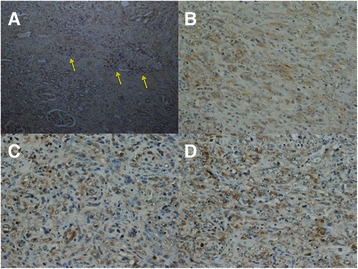
**Representative immunohistochemistry in the primary tumor tissues for anti-phosphorylated Akt (Ser-473), phosphorylated Akt (Thr-308), and phosphorylated S6 ribosomal protein (Ser-235/236) antibodies.** Some of tumor cells showed brown staining for anti-pAkt (Ser-473) antibody (arrows), while glomerulus and renal tubules did not (**A**. ×100). Many of tumor cells showed moderate to strong brown staining in a membrane and cytoplasm in RCC for anti-pAkt (Ser-473) antibody (**B**. ×200), for anti-pAkt (Thr-308) antibody (**C**. ×200), and for anti-pS6 antibody (**D**. ×200).

The amount of pAkt (Ser-473) pAkt (Thr-308) and pS6 protein was significantly greater in the primary tumors than in the non-tumor kidney tissues, defined as 1.0, (Figure 2, Table 2). Some of tumor cells showed brown staining in a membrane and cytoplasm, while glomerulus and renal tubules did not (Figure 3).

**Table 2:** **Relationship between SUVmax and pAkt/pS6K protein densitometry results and pathological parameters**

		SUVmax in the primary tumor			Western blotting in primary tumor						
		Number=77			Number=77	pAkt (Ser-473)		pAkt (Thr-308)		pS6K (Ser-235/236)	
			mean ± S.D	P value		mean ± S.D	P value	mean ± S.D	P value	mean ± S.D	P value
Tissue	tumor	n=77	6.97 ± 5.96		n=77	3.91 ± 3.01	0.000001	2.83 ± 2.13	0.000009	2.43 ± 1.58	0.000021
	non-tumor				n=77	1.00		1.00		1.00	
Histological differentiation (Fuhrman grade)	G1	n=9	2.72 ± 0.66	0.000073	n=9	1.54 ± 0.57	0.000041	1.21 ± 0.36	0.000089	1.13 ± 0.15	0.000079
	G2	n=32	3.90 ± 2.33		n=32	2.57 ± 2.03		2.03 ± 1.57		1.86 ± 1.08	
	G3	n=29	8.80 ± 3.79		n=29	5.16 ± 2.73		3.46 ± 1.85		2.81 ± 1.23	
	G4	n=7	18.67 ± 10.23		n=7	8.09 ± 3.49		6.00 ± 2.71		5.19 ± 2.07	
pT stage	pT1-2	n=43	3.77 ± 2.02	0.000019	n=43	2.43 ± 1.74	0.000029	1.93 ± 1.49	0.000083	1.75 ± 0.95	0.000100
	pT3-4	n=34	10.91 ± 6.82		n=34	5.83 ± 3.24		4.00 ± 2.27		3.31 ± 1.79	
Microscopic vascular invasion	v0	n=28	3.33 ± 1.46	0.000061	n=28	2.08 ± 1.22	0.000100	1.67 ± 1.14	0.000200	1.60 ± 0.78	0.001000
	v1	n=49	9.01 ± 6.55		n=49	4.97 ± 3.23		3.50 ± 2.28		2.91 ± 1.72	
pN stage	N0	n=62	5.58 ± 5.80	0.000053	n=62	3.13 ± 2.54	0.000076	2.41 ± 2.00	0.000200	2.04 ± 1.31	0.000092
	N1-2	n=15	12.38 ± 2.59		n=15	7.38 ± 2.45		4.68 ± 1.64		4.14 ± 1.56	
Metastasis (cM stage)	M0	n=52	4.56 ± 3.59	0.000029	n=52	2.55 ± 2.03	0.000043	2.07 ± 1.69	0.000085	1.81 ± 1.52	0.000078
	M1	n=25	12.10 ± 6.78		n=25	7.04 ± 2.53		4.57 ± 2.03		3.85 ± 1.16	
cell histology	clear cell carcinoma	n=57	4.94 ± 3.15	0.000069	n=57	3.16 ± 2.44	0.000500	2.28 ± 1.68	0.000300	2.03 ± 1.19	0.001300
	non-clear cell carcinoma	n=11	9.44 ± 5.04		n=11	4.43 ± 3.36		3.21 ± 2.11		3.41 ± 2.24	
	Sarcomatoid*	n=9	16.54 ± 9.37		n=9	8.56 ± 1.88		6.17 ± 2.02		3.95 ± 1.59	

Data show mean ± S.D.

Sarcomatoid*: clear cell carcinoma with sarcomatoid differentiation.

Karnofsky performance status below 80% (n=19) showed higher p-SUVmax than that above 80% (n=58) (13.61 ± 7.47 vs 4.84 ± 3.29, *P* = 0.000052).

**Figure 4:**
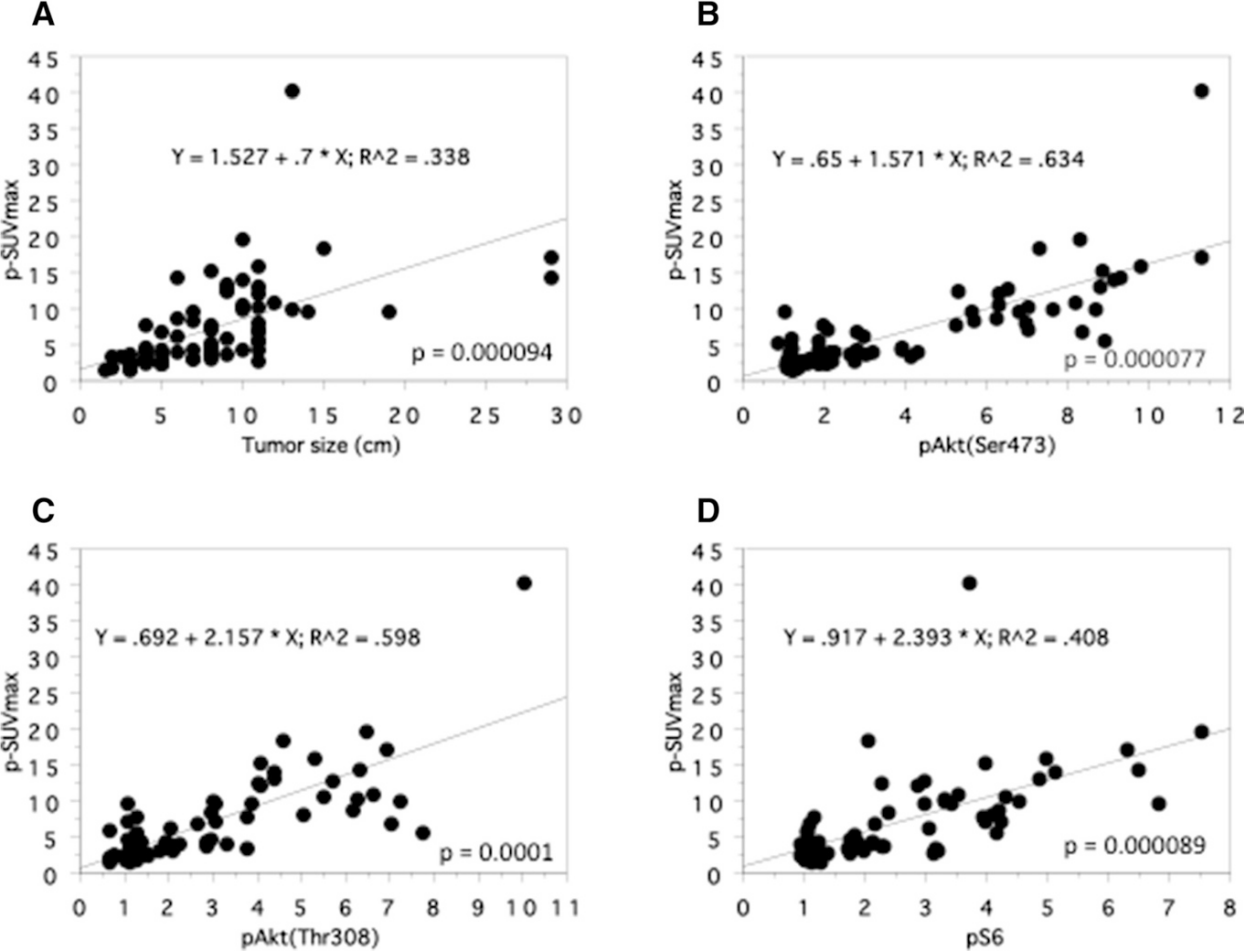
**Spearman rank correlation between the SUVmax and the tumor size and the expression levels of phosphorylated proteins in the primary tumors (n=77). A**: tumor size. **B**: pAkt (Ser-473). **C**: pAkt (Thr-308). **D**: pS6.

We investigated the correlation between SUVmax of the primary tumors (p-SUVmax) and tumor size. When tumor size was set as the independent variable and p-SUVmax as the dependent variable, a positive correlation was observed (r^2^ = 0.34, *P* = 0.000094, Figure 4A).

**Figure 5:**
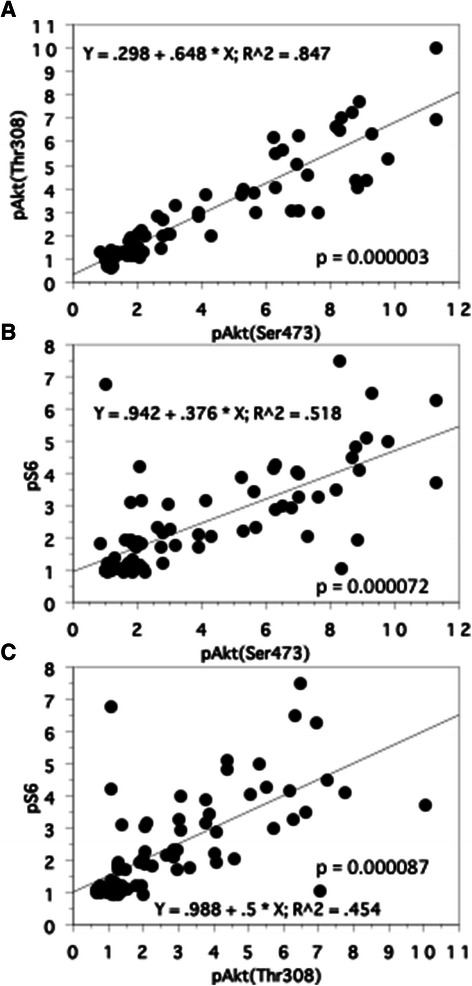
**Spearman rank correlation between the expression levels of phosphorylated proteins in the primary tumors (n=77). A**: pAkt (Ser-473) and pAkt (Thr-308). **B**: pAkt (Ser-473) and pS6. **C**: pAkt (Thr-308) and pS6.

Regarding with the correlation between p-SUVmax and the expression of pAkt (Ser-473), pAkt (Thr-308), and pS6 proteins in the primary tumors, a positive correlation was observed (pAkt (Ser-473); r^2^ = 0.63, *P* = 0.000077, pAkt (Thr-308); r^2^ = 0.60, *P* = 0.0001, pS6; r^2^ = 0.41, *P* = 0.000089, Figures 4B-D). There was also a positive correlation between pAkt (Ser-473) and pAkt (Thr-308) (r^2^ = 0.85, *P* = 0.000003, Figure 5A), pAkt (Ser-473) and pS6 (r^2^ = 0.52, *P* = 0.000072, Figure 5B), and pAkt (Thr-308) and pS6 (r^2^ = 0.45, *P* = 0.000087, Figure 5C).

### Higher SUVmax and elevated expression of pAkt and pS6 of the primary tumor was associated with its aggressive and metastatic profiles

A higher p-SUVmax was related to less tumor differentiation, local invasion, regional lymph node involvement, microscopic vascular invasion, distant metastasis, and non-clear cell histology (non-ccRCC) (Table 2).

Similarly, increased expression of pAkt (Ser-473) pAkt (Thr-308), and pS6 in the primary tumors was correlated with less differentiation, local invasion, regional lymph node involvement, microscopic vascular invasion, distant metastasis, and non-ccRCC (Table 2).

Regarding with the relationship between the p-SUVmax and the highest SUVmax of the metastatic tumors (m-SUVmax), there was a tendency toward correlation (r^2^ = 0.17, *P* = 0.0568). On the other hand, the highest m-SUVmax was not significantly correlated with the expression of pAkt (Ser-473) (r^2^ = 0.04, *P* = 0.3835), pAkt (Thr-308) (r^2^ = 0.05, *P* = 0.3526), or pS6 in the primary tumor (r^2^ = 0.01, *P* = 0.9803).

### A higher SUVmax and increased expression of pAkt and pS6 of the primary tumor was associated with early relapse following radical nephrectomy and with shorter overall survival

**Figure 6:**
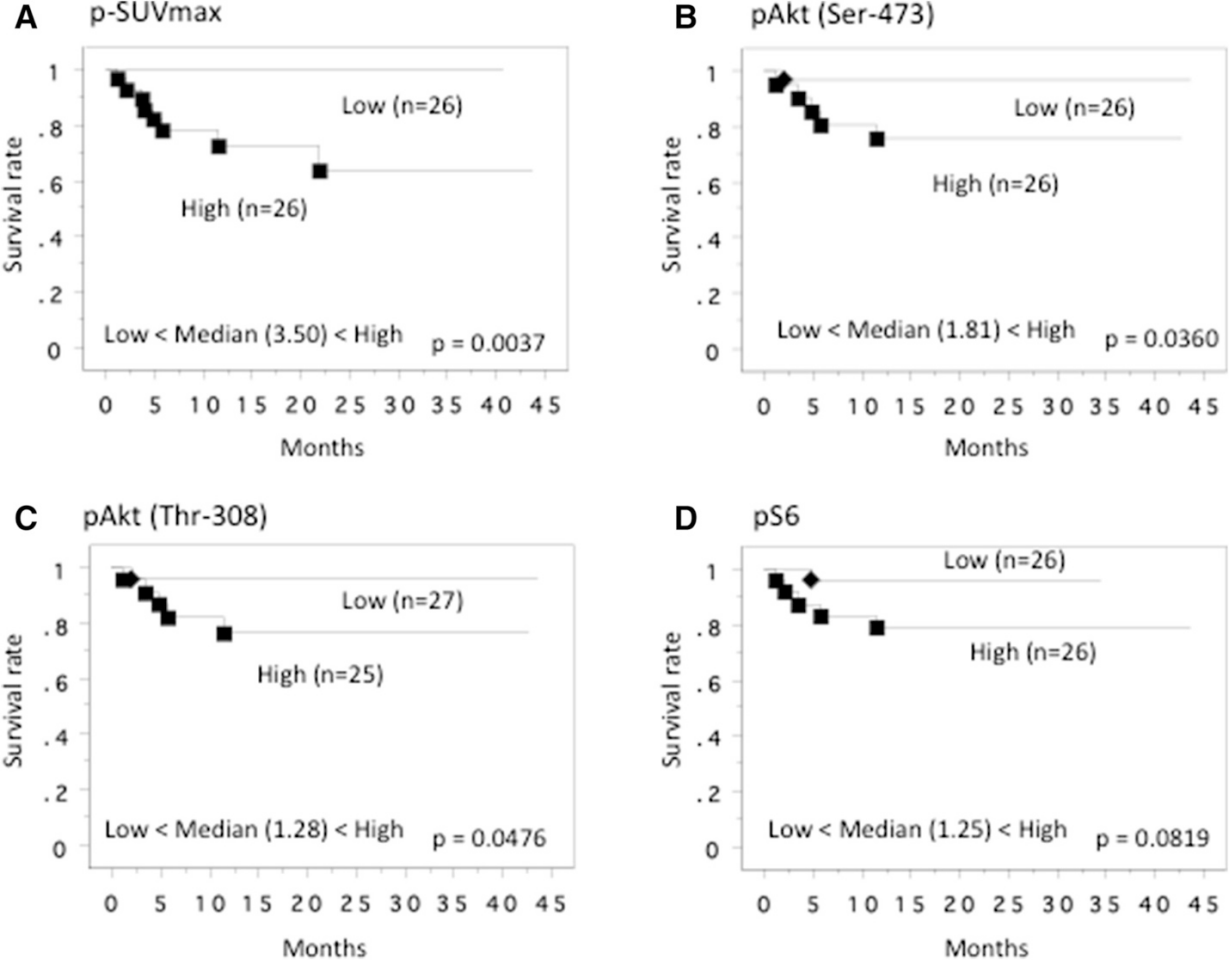
**Recurrence free-survival curve in 52 M0 cases at nephrectomy.** This survival curve is based on the median values of SUVmax **(A)** and protein expression of pAkt(Ser-473) **(B)**, pAkt(Thr-308) **(C)** and pS6 proteins **(D)** in the primary tumor. The cases were divided into two groups at this levels - high and low expression. *P* value was analyzed by log-rank test.

When the 52 patients with M0 tumors were divided into two groups at the median p-SUVmax (3.50), comparison of the Kaplan-Meier survival rate plots with low vs. high p-SUVmax value linked high p-SUVmax with early relapse after nephrectomy (Figure 6A). Tumors with higher pAkt (Ser-473) and pAkt (Thr-308) expression groups in the primary tumors is associated with early relapse after nephrectomy, but pS6 did not (Figures 6B-D). While less differentiation, local invasion, microscopic vascular invasion, non-clear cell, sarcomatoid differentiation, higher p-SUVmax, higher pAkt (Ser-473), and higher pAkt (Thr-308) were significant by Cox univariate analysis, only less differentiation was significant by multivariate analysis (Table 3).

**Table 3:** **Cox regression analysis for various potential prognostic factors in recurrence-free survival**

			Recurrence-free survival in cN0M0 cases			
Variable	Unfavorable/favorable characteristics	No. of Patients	Analysis	Relative risk	95% confidential interval	P value
p-SUVmax*	**high/low**	**26/26**	**Univariate (U)**	**14.361**	**4.283 - 64.472**	**0.00940**
			**Multivariate (M)**	**1.714**	**0.021 - 142.351**	**0.64270**
**pAkt (Ser473)**	**high/low**	**26/26**	**U**	**5.895**	**1.688 - 50.514**	**0.03910**
			**M**	**2.374**	**0.062 - 367.647**	**0.78520**
**pAkt (Thr308)**	**high/low**	**25/27**	**U**	**5.321**	**1.621 - 45.612**	**0.04810**
				**2.157**	**0.069 - 397.026**	**0.79130**
**pS6**	**high/low**	**26/26**	**U**	**3.994**	**0.466 - 34.272**	**0.12190**
			**M**			
**Grade**	**4/3/2/1**	**2/13/28/9**	**U**	**11.397**	**3.320 - 33.119**	**0.00007**
			**M**	**31.171**	**1.836 - 529.228**	**0.01730**
**pT**	**4,3/2,1**	**13/39**	**U**	**7.807**	**1.920 - 31.753**	**0.00410**
			**M**	**5.151**	**0.187 - 151.279**	**0.15370**
**v**	**1/0**	**24/28**	**U**	**4.941**	**1.016 - 24.035**	**0.04780**
			**M**	**2.355**	**0.156 - 35.559**	**0.53630**
**cell type**	sarcomatoid/non-cc/cc*	**3/9/40**	**U**	**7.051**	**2.634 - 18.876**	**0.00010**
			**M**	**1.029**	**0.091 - 11.577**	**0.78180**

p-SUVmax*: SUVmax value in the primary tumor.

sarcomatoid/non-cc/cc*: sarcomatoid differentiation/non-clear cell RCC/clear cell RCC.

**Figure 7:**
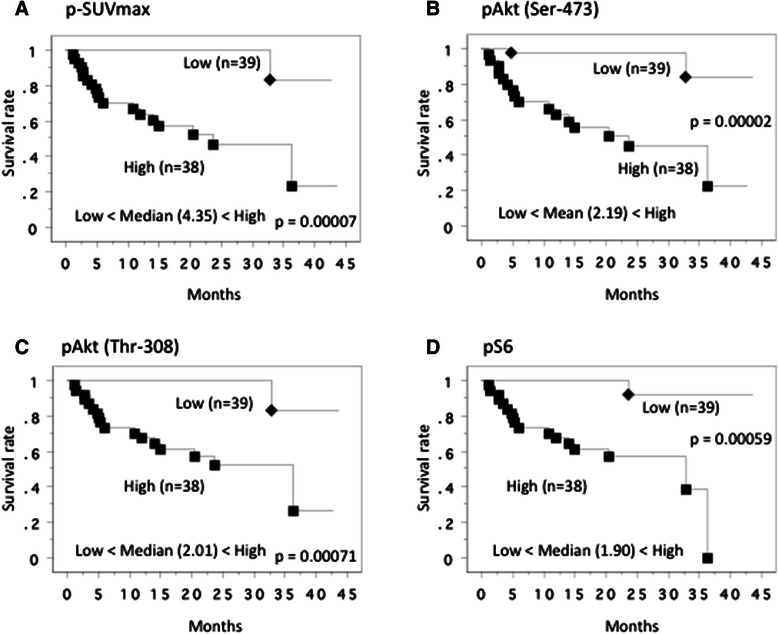
**Overall survival curve in all patients.** This survival curve is based on the median values of SUVmax **(A)** and protein expression of pAkt(Ser-473) **(B)**, pAkt(Thr-308) **(C)** and pS6 proteins **(D)** in the primary tumor. The cases were divided into two groups at this levels - high and low expression. *P* value was analyzed by log-rank test.

Regarding with the overall survival, the median level of p-SUVmax was 4.35, so the patients were divided into two groups at this cut-off value. Kaplan-Meier plots of survival for the higher and lower value groups showed that the higher p-SUVmax was associated with shorter overall survival (Figure 7A). Similarly, Kaplan-Meier plots showed that higher pAkt (Ser-473), pAkt (Thr-308) and pS6 expression levels in the primary tumors were associated with shorter overall survival (Figures 7B-D).

**Figure 8:**
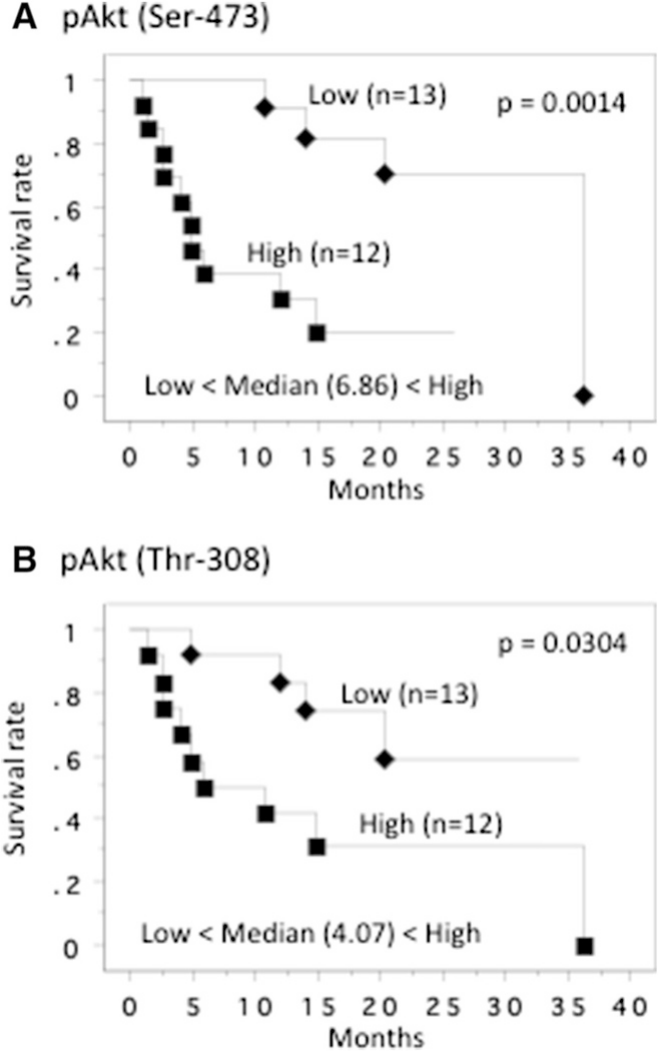
**Overall survival curve in 25 metastatic tumors at nephrectomy.** This survival curve is based on the median values of protein expression of pAkt(Ser-473) **(A)** and pAkt(Thr-308) **(B)**, the cases were divided into two groups at this levels - high and low expression. *P* value was analyzed by log-rank test.

We divided the M1 cases at nephrectomy (25 patients) into two groups in order to examine the relation of m-SUVmax, p-SUVmax, pAkt (Ser-473), pAkt (Thr-308) and pS6 expression levels in the primary tumors with the survival time. The median value of m-SUVmax in M1 cases was 7.30, so the patients were divided into two groups at this cut-off value to give a higher value group (n=13) and a lower value group (n=12). The patients with higher m-SUVmax had a tendency toward shorter overall survival (*P* = 0.0559). We also calculated the median value of p-SUVmax of the M1 cases at nephrectomy (25 patients), then those were divided into two groups at median value. Kaplan-Meier plots of survival for patients with low vs. high p-SUVmax showed no statistic difference (*P* = 0.2791) for overall survival time. Similarly, regarding of the pAkt (Ser-473), pAkt (Thr-308) and pS6 expression levels in the primary tumors, we divided 25 M1 cases into two groups. The patients with higher pAkt (Ser-473) and pAkt (Thr-308) expression showed a shorter overall survival time (*P* = 0.0012, *P* = 0.0304, respectively, Figure 8), but pS6 had no impact (*P* = 0.2808).

## Discussion

RCC is characterized by impaired oxidative phosphorylation and a shift to aerobic glycolysis, which is a form of metabolic reprogramming known as cancer cell glycolysis (or the Warburg effect). An increase of glycolysis generates the adenosine triphosphate (ATP) needed for rapid proliferation and also enhances fatty acid synthesis by diminishing the phosphorylation of acetyl CoA carboxylase (a rate-limiting step of fatty acid synthesis) in order to provide energy for rapid tumor growth [7]. ^18^F-FDG PET has been widely used to detect malignancy and predict the prognosis, as well as being employed for tumor staging/restaging and in therapeutic decision-making and monitoring [10,11].

The aim of the present study was to investigate whether ^18^F-FDG PET could be used for noninvasive assessment of the biological characteristics of human RCC by comparing the relevance of SUVmax with clinicopathological features in human RCCs from a molecular point of view. In this study, we investigated the following: 1) relationship between p-SUVmax and expression of pAkt (Ser-473), pAkt (Thr-308), and pS6 protein in the primary tumor; 2) whether the p-SUVmax predicted relapse of organ-confined or locally advanced RCC without distant metastasis (cT_any_N_any_M0) after radical nephrectomy; and 3) whether the p-SUVmax or m-SUVmax predicted the response of metastatic lesions (cT_any_N_any_ M1) to cytokine/immunotherapy and/or molecular targeting therapy after radical nephrectomy.

### Relationship between SUVmax of the primary tumor and clinicopathological features

The p-SUVmax was positively correlated with Karnofsky performance status and tumor size, and was also correlated with less tumor differentiation, local invasion, regional lymph node involvement, microscopic vascular invasion, distant metastasis, and non-ccRCC histology. Furthermore, the p-SUVmax was positively correlated with the expression of pAkt (Ser-473), pAkt (Thr-308), and pS6 protein in the primary tumor, and the levels of these protein were also closely related to less differentiation, local invasion, regional lymph node involvement, microscopic vascular invasion, distant metastasis, and non-ccRCC histology.

Overactivation of the PI3K-Akt-mTOR pathway has been reported in various human cancers, including RCC [9]. mTOR forms mTOR complex (mTORC)1 and mTORC2 by binding to the regulatory associated protein of mTOR (Raptor) and the rapamycin-insensitive companion of mTOR (Rictor), respectively, and these two complexes have different intracellular functions. mTORC1 is activated by PI3K-Akt and it phosphorylates S6K1 and 4EBP1, thereby promoting translation and protein synthesis. Both this study and our previous investigations showed that pS6, the best-characterized downstream effector of mTORC1, is upregulated in primary renal tumors with metastasis [16,17], indicating that pS6 might influence the progression of RCC.

mTORC2 regulates the actin cytoskeleton and also possesses PDK2 activity that phosphorylates Ser-473 at the carboxy-terminus of Akt, which is essential for activation of Akt [25]. Activation of Akt may increase cell viability after inhibition of mTORC1, or could potentially the production of increase vascular endothelial growth factor (VEGF) because PI3K/Akt signaling induces tumor angiogenesis by regulating VEGF via both HIF1α-dependent and -independent mechanisms [26]. It has been reported that HIF1α expression is dependent on both raptor and rictor, whereas HIF2α expression only depends on rictor, with HIF2α and mTORC2 being more important in RCC [8,27]. We previously reported that tumors showing higher expression of pAkt (Ser-473) protein were resistant to treatment with interferon alpha and sorafenib, and that higher tumor levels of pAkt (Ser-473) were associated with shorter overall survival [16]. Similarly, Jonasch et al. reported that an increase of pAkt (Ser-473) expression revealed by microarray analysis was associated with worse survival of patients treated with sorafenib and interferon alpha [28]. Furthermore, we recently reported that assessing the expression of pAkt (Ser-473) in resected specimens might be useful for predicting the response of locally advanced RCC to neoadjuvant therapy with axitinib [23]. These findings suggest that pAkt (Ser-473) might be a key molecule in the progression of RCC and could be a potential biomarker for assessing the efficacy of targeted inhibition of the PI3K/Akt pathway. However, the role of pAkt (Thr-308) in RCCs has not been fully elucidated. So, we examined the expression for both pAkt (Ser-473) and pAkt (Thr-308) by co-expression analysis using surgically resected samples.

In the present study, increased expression of pAkt (Thr-308) in the primary renal tumor was also correlated with aggressive biological behavior and metastatic potential. Furthermore, expression of pAkt (Ser-473) and pAkt (Thr-308) in primary tumor tissue showed a strong positive correlation. Phosphorylation at two sites is required for full activation of Akt, since it is phosphorylated by PI3K-dependent kinase-1 (PDK1) at a threonine residue in the catalytic domain (Thr-308) and by PI3K-dependent kinase-2 (PDK2) at a serine residue (Ser-473) located in the carboxy-terminal hydrophobic motif [29]. Taken together, activation of both of pAkt (Ser-473) and pAkt (Thr-308) might be very important in the progression of RCC. Because SUVmax was positively correlated with the expression of pAkt (Ser-473), pAkt (Thr-308) and pS6, ^18^F-FDG PET might be a useful imaging modality for assessing the biological characteristics of RCC from a molecular point of view.

### Usefulness of SUVmax of the primary tumor for predicting the prognosis

Tumors with a higher p-SUVmax showed earlier relapse after radical nephrectomy. The tumors with early relapse also displayed higher expression of pAkt (Ser-473), pAkt (Thr-308), and pS6. These findings suggest that the patients with a higher SUVmax of the primary renal tumor should be under active surveillance, even if the tumor is well or moderately differentiated or is a noninvasive T1 lesion.

As is often the case with the patients with metastatic RCCs who received systemic therapy, while the sizes and/or the numbers of some metastatic lesions decreased, those of other lesions increased or new lesions appeared. Furthermore, some of the patients who showed poorer response for first-line systemic therapy for metastatic lesions had sensitivity for second-line systemic therapy. These might help to explain why many patients with metastatic disease show better survival when they receive sequential therapy with multiple targeting agents and current best supportive care than was achieved with conventional immuno-cytokine therapy [9]. It is likely that the biological characteristics of the primary and metastatic tumors were not always identical. Gerlinger et al. reported that primary and metastatic lesions showed biological differences and suggested that intra-patient tumor heterogeneity meant treatment strategies should be re-considered on the basis of these differences [30]. Thus, we should not select the treatment strategy by assuming that the characteristics of metastatic tumors are the same as those of the primary tumor. Since we could not obtain samples of every metastasis, however, we had to predict the biological characteristics of the metastatic lesions by examining the surgically resected primary tumors. In the present study, there was a correlation between p-SUVmax and the highest m-SUVmax of the metastatic tumors, as well as between p-SUVmax and the expression of pAkt (Ser-473), pAkt (Thr-308), and pS6 protein in the primary tumor, indicating that the metastatic tumors with higher m-SUVmax might have higher protein expression for pAkt (Ser-473), pAkt (Thr-308) and pS6.

It has also been reported that a decrease of SUVmax is associated with the response to various anticancer therapies [11], and use of ^18^F-FDG-PET as a pharmacodynamic biomarker for assessing the efficacy of inhibiting the PI3K/Akt pathway has been proposed [10]. Namura et al. reported that the survival of patients can be predicted by evaluating the SUVmax of metastatic tumors using ^18^F-FDG PET [31]. In addition, Ueno et al. reported that ^18^F-FDG PET/CT could be used to evaluate the early response of metastatic RCC to treatment with tyrosine kinase inhibitors (TKIs), since tumor size and FDG uptake on ^18^F-FDG PET/CT after 1 month of treatment predicted progression-free survival and overall survival [13]. These findings suggest that the SUVmax of metastatic tumors might be a biomarker that provides useful information for clinical decision making. However, in Japan, ^18^F-FDG PET is approved for one examination as a method of staging RCC, but repeat imaging to evaluate the treatment effect is not covered by the Japanese health insurance system. Therefore, we could not examine the influence of radical nephrectomy or systemic treatment on metastatic lesions by repeating ^18^F-FDG PET. Accordingly, we should make efforts to assess the biological characteristics of metastatic tumors from data obtained by ^18^F-FDG PET prior to nephrectomy.

Regarding with 25 metastatic (M1) cases, the m-SUVmax, p-SUVmax, pAkt (Ser-473), pAkt (Thr-308) and pS6 expression levels in the primary tumors were not associated with the response for first-line systemic therapy (data not shown). When we divided the 25 M1 cases into two groups at median value of p-SUVmax at nephrectomy, Kaplan-Meier plots of survival for patients with low vs. high p-SUVmax showed no statistic difference for overall survival time, while the patients with higher m-SUVmax had a tendency toward shorter overall survival, suggesting that the SUVmax of each metastatic lesion is important for predicting the biological characteristics. The patients with higher pAkt (Ser-473) and pAkt (Thr-308) expression showed a shorter overall survival time, but pS6 had no impact. On the other hand, when we analyzed all 77 patients, the aggressive and metastatic potential with higher p-SUVmax and pAkt (Ser-473), pAkt (Thr-308) and pS6 expression levels in the primary tumors were associated with unfavoravle prognosis. Taken together, SUVmax, pAkt (Ser-473), pAkt (Thr-308) and pS6 might be biological markers in RCCs.

The present study was not randomized and evaluated a relatively small number of patients, while the follow-up period was too short to draw definite conclusions. Immunohistochemical study showed the positive staining in membrane and cytoplasm in cancer cells, but not in normal glomerulus and renal tubules. Although these findings supported the data obtained by Western blotting, we should investigate the relationship between SUVmax and the staining intensity and localization of pAkt (Ser-473), pAkt (Thr-308) and pS6. On the other hand, glucose transporters are responsible for ^18^F-FDG uptake by cancer cells and GLUT1 expression has been positively correlated with ^18^F-FDG uptake [32]. GLUT1 is thought to be a possible intrinsic marker of hypoxia, and it has been reported that GLUT1 expression is regulated by hypoxia via hypoxia inducible factor (HIF)-1 [33]. Upregulation of the HIF-1 pathway has been identified in RCC, and hypoxia is associated with higher ^18^F-FDG uptake [12]. HIF-1 is considered to support tumor growth by induction of angiogenesis via increased expression of VEGF and also by promoting anaerobic metabolism [34]. Akt inhibition disrupts transcription of GLUT1 and its translocation to the plasma membrane to promote glucose utilization independent of an effect on cell proliferation [15]. Accordingly, we should comprehensively investigate the role of HIF-1, GLUT1, Akt in RCC in the future. Obtaining such information may be important to elucidate the relationship between clinicopathological features and molecular changes associated with SUVmax, and will shed light on the clinical issue of whether ^18^F-FDG PET is a useful modality for therapeutic decision-making and monitoring.

## Conclusions

A higher SUVmax on ^18^F-FDG PET is associated with elevated tumor levels of pAkt and pS6 protein and with aggressive behavior and metastatic potential of RCC, as well as with early relapse following radical nephrectomy and shorter overall survival. These findings suggest that SUVmax may be useful for predicting the biological characteristics of RCC.
